# Optimization of GFP Fluorescence Preservation by a Modified uDISCO Clearing Protocol

**DOI:** 10.3389/fnana.2018.00067

**Published:** 2018-08-15

**Authors:** Yusha Li, Jianyi Xu, Peng Wan, Tingting Yu, Dan Zhu

**Affiliations:** ^1^Britton Chance Center for Biomedical Photonics, Wuhan National Laboratory for Optoelectronics, Huazhong University of Science and Technology, Wuhan, China; ^2^MoE Key Laboratory for Biomedical Photonics, Collaborative Innovation Center for Biomedical Engineering, School of Engineering Sciences, Huazhong University of Science and Technology, Wuhan, China

**Keywords:** tissue optical clearing, whole-brain imaging, green fluorescent protein, fluorescence preservation, uDISCO, a-uDISCO

## Abstract

Tissue optical clearing techniques provide alternative approaches for imaging large-volume specimens. uDISCO, an organic-solvent-based method, stands out from the enormous array of available optical clearing methods by achieving whole-brain imaging with high transparency, size reduction and fluorescence preservation. In this study, we aimed to modify the uDISCO protocol to achieve better fluorescence preservation and to thereby further improve its optical imaging quality. First, we determined the optimal pH value for optimized uDISCO, termed “a-uDISCO” (alkaline pH-based uDISCO). Then, we compared fluorescence preservation between a-uDISCO and uDISCO. In addition, we validated the clearing performance of the optimized method according to several parameters, including tissue transparency, size changes, and the maintenance of cell morphology. Finally, we demonstrated that a-uDISCO enabled the high-quality brain-wide visualization of neuronal structures. This method potentially provides a better alternative for high-throughput imaging of samples with low-level fluorescence protein expression or for archiving and repetitive revisiting of rare samples.

## Introduction

Whole-brain imaging at single-cell resolution has become indispensable for understanding brain structure-function relationships. Conventional histological methods based on thin sections are commonly used but time-consuming, labor-intensive and prone to information loss. Automated sectioning methods, such as micro-optical sectioning tomography (MOST) and serial two-photon tomography (STP), can eliminate information loss and have been successfully used to obtain high-resolution images throughout the entire brain (Li et al., 2010; Gong et al., 2013; Amato et al., 2016). However, these mechanical sectioning-based methods can destroy sample tissues.

The emergence of the tissue optical clearing (TOC) technique in combination with the development of light-sheet microscopy has provided an alternative approach for three-dimensional (3D) imaging of the whole brain (Tuchin, 2005; Osten and Margrie, 2013; Zhu et al., 2013; Liu et al., 2017; Yu et al., 2018). Currently, various optical clearing methods have been developed, and these methods homogenize the differences in refractive indices between different biological components by immersing the tissue in an appropriate agent to reduce scattering and transform intact tissue into optically transparent tissue samples (Yu et al., 2011; Richardson and Lichtman, 2015). They are mainly divided into two types, including aqueous-based clearing methods and solvent-based clearing methods. The former includes Sca*l*e, Sca*l*eS, SeeDB, SeeDB2, CUBIC, CLARITY, and OPTIClear (Hama et al., 2011, 2015; Chung et al., 2013; Ke et al., 2013, 2016; Susaki et al., 2014; Lai et al., 2018). These aqueous-based clearing methods either increase or do not change the size of the tissues, while the organic solvent-based clearing methods, such as 3DISCO, FluoClearBABB and uDISCO, can reduce sample size and provide a high level of transparency, which is useful for imaging large volumes (Ertürk et al., 2012; Schwarz et al., 2015; Pan et al., 2016).

In recent decades, the green fluorescent protein (GFP) has been a valuable fluorescent marker for labeling specific proteins in cell biology (Tsien, 1998). A great number of GFP-expressing transgenic mouse lines containing the selective expression of GFP in specific cells have already been generated and are commonly used in neuroscience studies (Feng et al., 2000). Hence, the preservation of GFP fluorescence during the clearing process is just as essential as achieving high transparency and size reduction when used for large-volume imaging. However, some solvent-based clearing methods, such as 3DISCO, quickly quench GFP (Ertürk and Bradke, 2013). The more recently developed uDISCO method can achieve a relatively high level of GFP fluorescence preservation over the accompanying inherently strong background fluorescence, which could decrease the signal-to-noise ratio of fluorescence images (Pan et al., 2016; Lai et al., 2017). Enhancing fluorescence preservation is an alternative way to improve image quality. Thus, it is necessary to optimize the GFP fluorescence-preserving capability of the uDISCO protocol.

In this study, we aimed to develop a modified protocol for better fluorescence preservation based on uDISCO. First, we adjusted the pH values of clearing agents and determined the optimal pH value for GFP stability so that we could modify uDISCO, with the new protocol termed a-uDISCO. Then, we quantitatively evaluated the fluorescence-preserving capability of a-uDISCO treatment for short-term and long-term storage and compared them with uDISCO treatment. Additionally, we investigated its clearing performance, including transparency, changes in size and cell morphology. Finally, we conducted 3D imaging of brain-wide neural networks by combining a-uDISCO with a light-sheet microscope.

## Materials and methods

### Animals

Adult *Thy1*-GFP-M line mice (2–3 months old) and *Cx3cr1*-GFP mice (2–3 months old) were used in this study (Jackson Laboratory, USA; RRID:IMSR_JAX:007788; RRID:IMSR_JAX:005582). All experimental procedures were performed in strict accordance with the Experimental Animal Management Ordinance of Hubei Province, China, and were approved by the Institutional Animal Ethics Committee of Huazhong University of Science and Technology.

### Sample preparation

Adult mice were deeply anesthetized with an intraperitoneal injection consisting of a mixture of 2% α-chloralose and 10% urethane (8 ml/kg) and then transcardially perfused with 0.01 M PBS (Sigma) followed by 4% paraformaldehyde (PFA, Sigma-Aldrich) in 0.01 M PBS. The brains and other organs were excised and post-fixed overnight at 4°C in 4% PFA. After PFA post-fixation, the samples were washed with 0.01 M PBS at least three times. Then, 1 mm-thick coronal brain sections were sliced on a vibratome (Leica VT 1000s). The hemispheres were separated by cutting the brains along the midline.

### Adjustment of PH value

pH values were measured with a pH meter [Ohaus instruments (Shanghai) co. LTD]. We tested the state of the glass electrode with calibration solutions before performing measurements. We continued measurements only when the pH meter showed a smiling or expressionless face. The reagent bottles were placed in a water bath to ensure that the temperature of the reagent was 25°C during all measurements.

### Clearing protocols

Tert-butanol (Sigma-Aldrich) solutions of increasing concentrations (30, 50, 70, 80, 90, 96, and 100 vol%) and a refractive index matching solution (BABB-D4) were prepared according to the original uDISCO protocol. BABB-D4 was prepared by mixing benzyl alcohol (Sigma-Aldrich), benzyl benzoate (Sigma-Aldrich) and diphenyl ether (Alfa Aesar) at a ratio of 4:8:3 (vol/vol/vol), and adding 0.4 vol% Vitamin E (Alfa Aesar). For protocols performed under different pH conditions, the pH values of the tert-butanol solution and BABB-D4 were adjusted to 7.5–8.0, 9.0–9.5, and 10.5–11.0 with using triethylamine (Sinopharm).

For all clearing protocols, the samples were processed as described in the original uDISCO study (Pan et al., 2016). Briefly, 1 mm-thick brain slices were serially dehydrated with graded tert-butanol solutions for 2 h/step (the 96 vol% tert-butanol step was performed overnight) and then cleared with BABB-D4 for 1 h at room temperature. The brain slices were stored in BABB-D4. For hemispheres or whole brains, the samples were serially dehydrated at 35°C through a graded series of tert-butanol solutions for the indicated times (Supplementary Table 1) and then immersed in dichloromethane (DCM, Sinopharm) for 45–60 min at room temperature to remove the lipids. Finally, hemispheres were cleared at room temperature in BABB-D4 for at least 3 h, while whole brains were cleared for 5 h. While the sample tissues were in storage, the refractive index matching solutions were replaced every 3–4 days.

Many of the reagents used in this study are toxic. The information of the reagents are listed in Supplementary Table 2. We performed all the experiments under a chemical hood except the imaging step.

### Measurement of light transmittance

The transmittance of whole brains was measured with a commercially available spectrophotometer (Lambda 950, PerkinElmer, USA). The whole brains were placed in a cuvette filled with the indicated refractive index matching solution for measurement. A small device was designed to hold the whole brain upright during measurement. This solution contained a silicon blob and plastic tubing (Figure 4B). A light beam oriented perpendicular to the transverse section of whole brains was passed through the central part of the brains, and the ventral-to-dorsal transmittance was then measured. The blank value was measured using a light beam oriented through a cuvette filled with the indicated refractive index matching solution without sample tissues. Each value was determined by averaging duplicate measurements.

### Imaging

**Figure 1:**
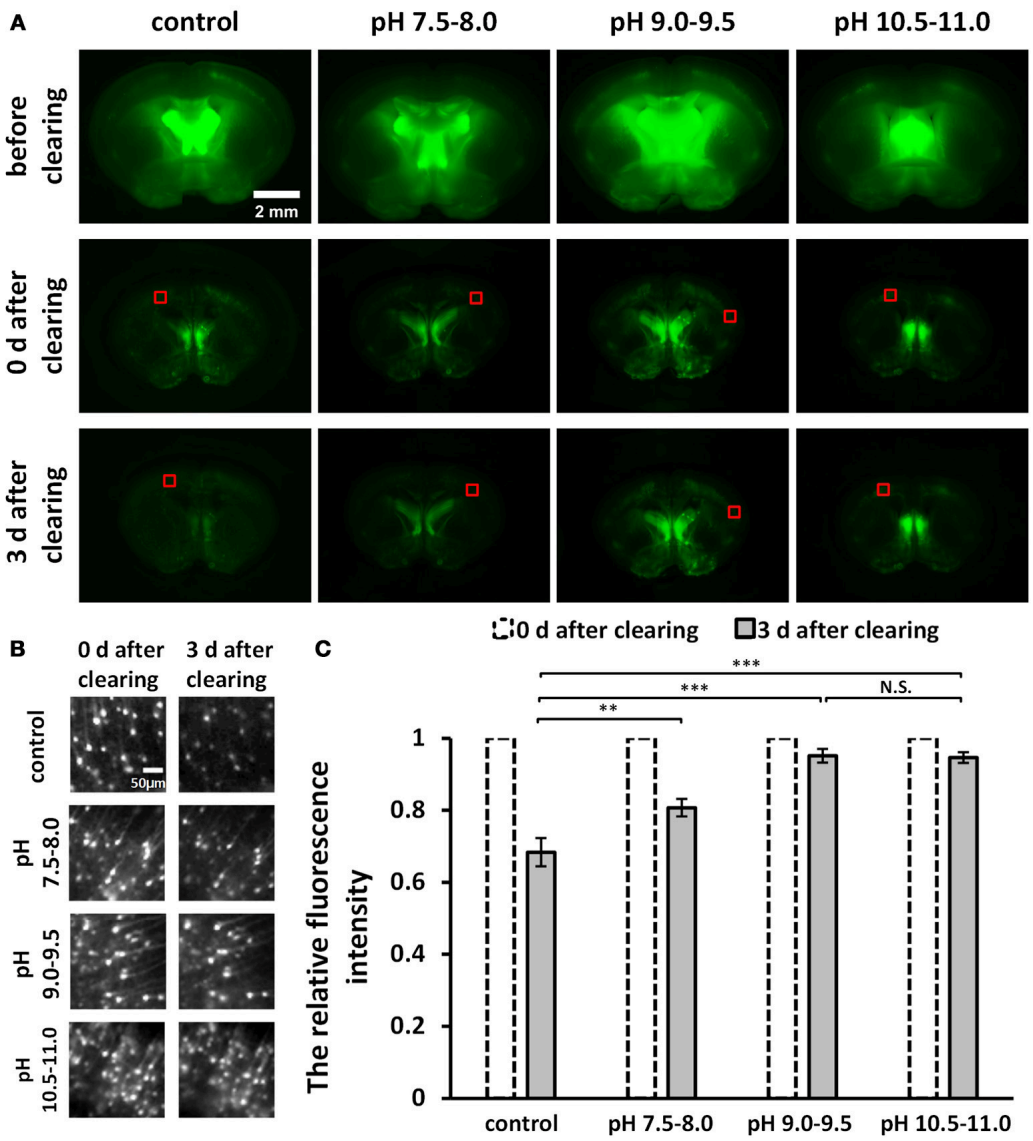
Screening for an optimal pH to optimize uDISCO for better fluorescence preservation (*Thy1*-GFP-M). **(A)** Fluorescence images of 1 mm-thick brain sections were obtained with a fluorescence stereomicroscope before and after clearing under different pH conditions. **(B)** Cropped fluorescence images of the cortex areas of 1 mm-thick brain sections, indicated in **(A)** with red boxes. **(C)** Relative mean fluorescence intensity of cortex areas of cleared brain slices (*n* = 7, 6, 8, and 8 areas for each condition). All values are shown as the mean ± s.e.m.; the statistical significance shown in **(C)** (N.S., not significant; ^**^P < 0.01; and ^***^P < 0.001) was assessed by One-way ANOVA followed by the least significant difference (LSD) test.

**Figure 2:**
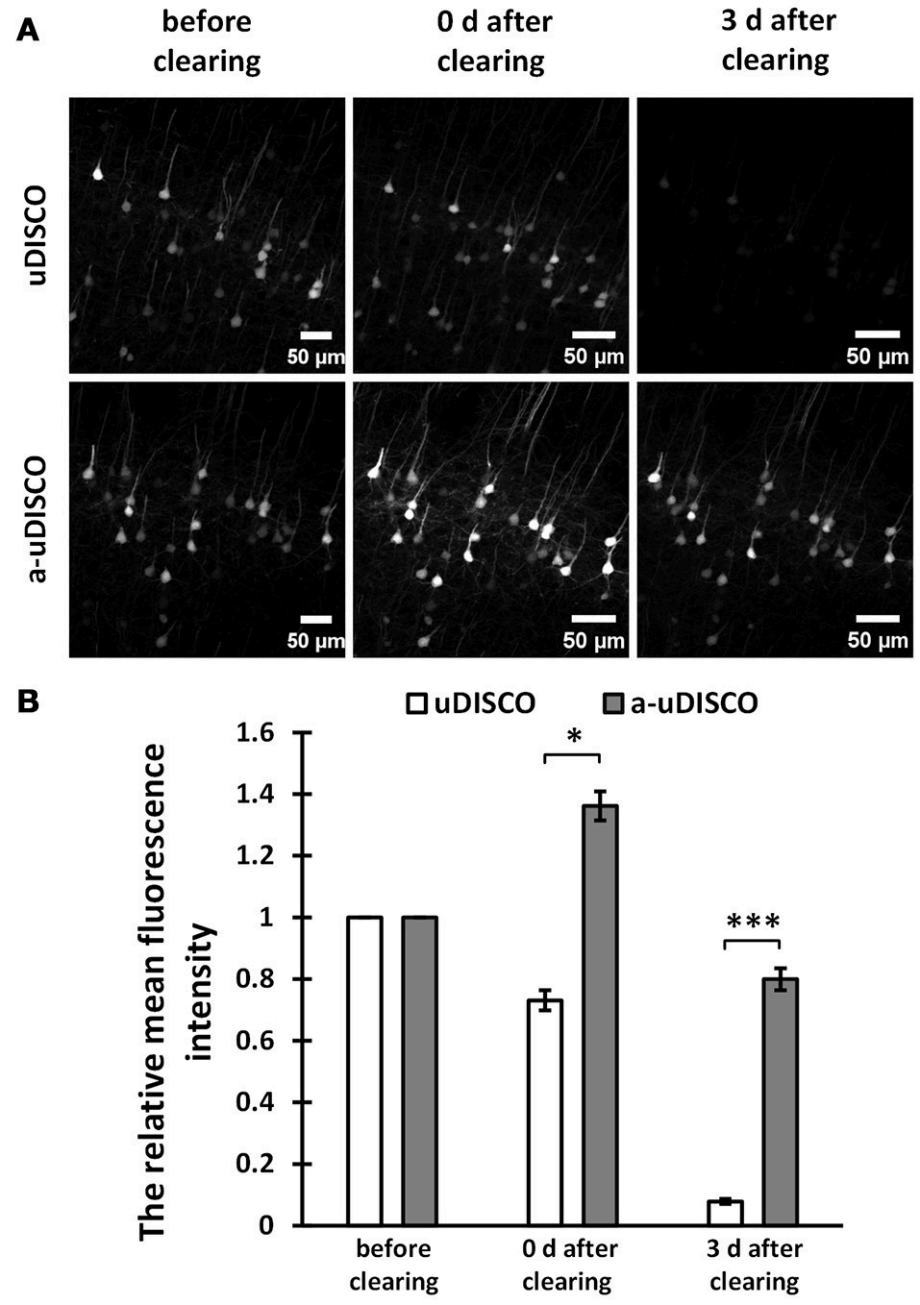
Quantitative comparison of fluorescence intensity in 1 mm-thick brain sections (*Thy1*-GFP-M). **(A)** Maximum intensity projections of the image stacks of corresponding neurons before and after clearing. **(B)** Quantification of the fluorescence intensity of corresponding neurons before and after clearing (*n* = 23 and 15 cell bodies in 6 brain slices, at day 0 and day 3 after cleared with uDISCO, respectively; *n* = 28 cell bodies in 6 brain slices, at day 0 and day 3 after cleared with a-uDISCO). All values are shown as the mean ± s.e.m.; the statistical significance in **(B)** (^*^P < 0.05; ^***^P < 0.001) was assessed by the unpaired two-tailed *t* test.

**Figure 3:**
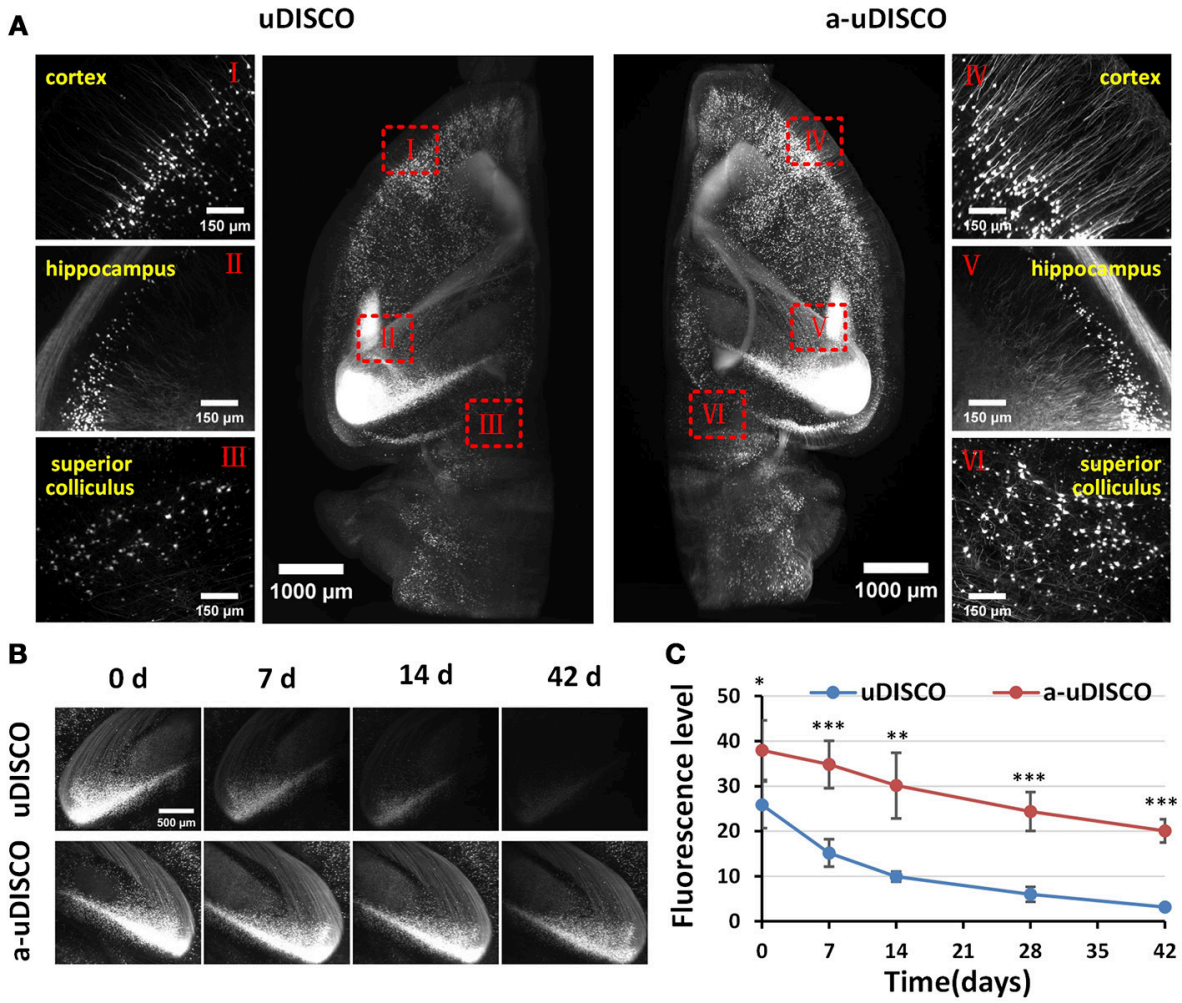
Comparison of fluorescence preservation during long-term storage of hemispheres (*Thy1*-GFP-M). **(A)** Light-sheet microscopy imaging of left and right hemispheres cleared with uDISCO and a-uDISCO, respectively. **(B)** High-magnification fluorescence images of the hippocampus at four time points during long-term storage after uDISCO and a-uDISCO showing the fluorescence decay of GFP. **(C)** Fluorescence-level quantification in hemispheres during long-term storage after uDISCO and a-uDISCO (*n* = 5 samples for each method). All values are shown as the mean ± s.d.; the statistical significance in C (^*^P < 0.05; ^**^P < 0.01; and ^***^P < 0.001) was assessed by the unpaired two-tailed *t* test.

Fluorescence images obtained using 1 mm-thick brain slices (Figure 1A) were acquired with a fluorescence stereomicroscope (Axio Zoom.V16, Zeiss, Germany) using a 2.3 × /0.57-long working distance air objective lens (W.D. 10.6 mm). The z-stack fluorescence images of neurons (Figure 2A) were acquired with an inverted confocal fluorescence microscope (LSM710, Zeiss, Germany) equipped with a Plan-Apochromat 10 × /0.5 dry objective (W.D. 2.0 mm). Fluorescence images of cleared hemispheres and whole brains were obtained by a light-sheet microscope (Ultramicroscope, LaVision BioTec, Germany) equipped with a 2 × /0.5 objective and a macro zoom body (magnification steps from 0.63 × to 6.3 ×). Three-dimensional images of entire hemispheres and whole brains (Figures 3A, 5A) were acquired with 0.8 × optical zoom, while high-magnification images (Figures 3A I–VI, 3B, 5B–F) showing neuronal structures at a subcellular resolution were acquired with 4.0 × optical zoom. When acquiring fluorescence images of samples cleared with different clearing protocols, we used the same acquisition settings.

**Figure 4:**
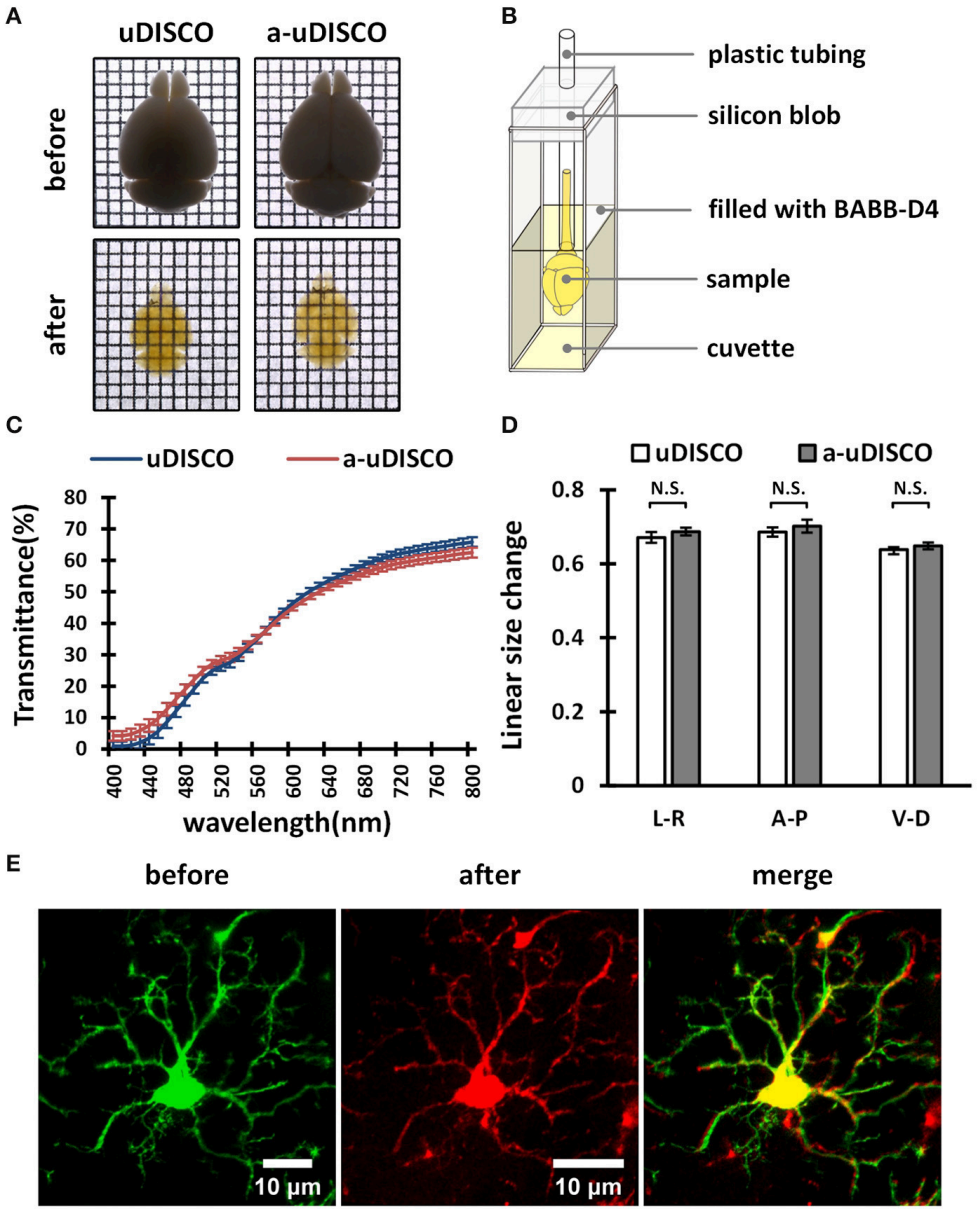
The performance of a-uDISCO for whole brain clearing. **(A)** Bright-field images of brains showing transparency after uDISCO and a-uDISCO. Grid size, 1.44 mm × 1.44 mm. **(B)** A small device held cleared brain upright for transmittance measurements. **(C)** The transmittance of the brains cleared with uDISCO and a-uDISCO (*n* = 3 samples for each method). **(D)** Size changes in whole adult mouse brains after uDISCO and a-uDISCO (*n* = 4 samples for each method). L–R, left side to right side; A–P, anterior side to posterior side; V–D, ventral side to dorsal side. **(E)** Cell morphology of microglia observed in whole mouse brains (*Cx3cr1*-GFP) before and after clearing by a-uDISCO. All values are shown as the mean ± s.e.m.; the statistical significance shown in **(D)** (N.S., not significant, *P* > 0.05) was assessed by the unpaired two-tailed *t* test.

The z-stack fluorescence images of individual microglia (*Cx3cr1*-GFP) (Figure 4E) and dendritic spines (*Thy1*-GFP-M) (Supplementary Figure 1) in whole adult mouse brains were acquired with an inverted confocal fluorescence microscope (LSM710, Zeiss, Germany) equipped with a Plan-Apochromat 40 × /1.4 oil objective (W.D. 0.13 mm) and an alphaPlan-Apochromat 63 × /1.46 oil objective (W.D. 0.1 mm). To ensure that we could find the same cell or dendrite after the sample was cleared, we imaged the map images using low-magnification lenses (5 × objective, 10 × objective and 20 × objective) (Supplementary Figure 2). In addition, we used the blood vessels as the anatomical landmarks to identify the same brain region during imaging.

About the imaging and clearing, we listed several potential problems and corresponding solutions in troubleshooting table (Supplementary Table 3).

### Image data processing

We used ImageJ software (RRID:SCR_003070) to analyze the images, and used Imaris (RRID:SCR_007370) for 3D reconstruction.

To roughly evaluate fluorescence decay during storage at different pH conditions, we cut out the equally sized images in the cortex (Figure 1B) from the wide-field fluorescence images of cleared samples in Figure 1A. We measured the mean intensity of cropped images and then calculated the relative fluorescence intensity by dividing the value obtained on day 3 after clearing by the value obtained on day 0 after clearing (Figure 1C).

For fluorescence quantification of 1 mm-thick brain slices (Figure 2B), we chose the image stacks at the surface of brain slices to obtain the maximum intensity projections (MIPs), and used the freehand selection function of ImageJ software to draw the outline of each cell body in MIP images (Supplementary Figure 3A), followed by measuring the mean intensity. Then we divided the value obtained after clearing by the value obtained before clearing to determine the relative mean fluorescence intensity.

To quantify fluorescence in hemispheres (Figure 3C), we chose a 30-μm z-projection of images in the cortex area to calculate the fluorescence level, which was regarded as the signal-to-background ratio (SBR), as described in the original uDISCO manuscript (Pan et al., 2016). Specifically, we used the threshold function of ImageJ software to identify the visible cell bodies and used “analyze particles” to select cell bodies with sizes between 100 and 500 μm^2^, and then measured the mean intensity of each selected cell body (Supplementary Figure 3B). The mean value of the signal was obtained by averaging the mean intensities of cell bodies. Then, we used the rectangle tool of ImageJ to select 15–20 background regions that showed no signal, and obtained the mean value of the background by averaging the mean intensities measured in these equally sized areas (20.31 × 20.31 μm). Finally, we calculated the fluorescence level by dividing the mean value of the signal by the mean value of the background.

### Statistical analysis

Statistical analyses were performed using SPSS software (RRID:SCR_002865). One-way ANOVA followed by the least significant difference (LSD) test was used to compare more than two groups (Figure 1C). The unpaired two-tailed t test was used to compare data between two groups (Figures 2–4).

## Results

### a-uDISCO: a modified clearing protocol based on uDISCO

To improve fluorescence preservation, we attempted to identify the optimal pH value for GFP stability that would optimize uDISCO. Here, for both the dehydration and the refractive index matching solutions (i.e., tert-butanol solutions and BABB-D4), we used triethylamine to adjust the pH value to several ranges, including 7.5–8.0, 9.0–9.5, and 10.5–11.0. We imaged 1 mm-thick brain sections obtained from *Thy1*-GFP-M mice under a fluorescence stereomicroscope both before and after clearing with uDISCO at different pH values. The images of cleared samples were acquired on day 0 and day 3 after the onset of clearing (defined as “0 d after clearing” and “3 d after clearing, respectively”), as shown in Figure 1A. We also evaluated fluorescence decay during storage by roughly calculating the relative fluorescence intensity of the cortical area on day 3 after clearing (Figure 1C). The results showed that pH values in the range of 9.0–9.5 and 10.5–11.0 provided better fluorescence preservation than those in the range of 7.5–8.0 and the unadjusted group.

Because pH values in the range of 10.5–11.0 require the addition of much more triethylamine (which can decrease transparency) to the clearing agents, we determined that pH values ranging from 9.0–9.5 were optimal for uDISCO, and we named this new protocol a-uDISCO (alkaline pH-based uDISCO).

### a-uDISCO achieves improved fluorescence preservation

To assess the GFP fluorescence-preserving capability of a-uDISCO, we quantitatively investigated fluorescence signals in brain sections and hemispheres before and after clearing using either a-uDISCO or uDISCO and then compared the results.

For 1 mm-thick brain sections, we imaged neurons in the cortex before and after clearing with a confocal microscope. The MIPs of z-stack images are shown in Figure 2A. The images of cleared samples were acquired on day 0 and day 3 after the onset of clearing. The relative mean fluorescence intensities were also calculated (Figure 2B). We found that a-uDISCO obtained approximately 136% of the mean fluorescence intensity immediately after clearing. This was significantly higher than that obtained using uDISCO (~73%). We also analyzed changes in fluorescence during short-term storage in refractive index matching solutions. After immersion in BABB-D4 for 3 days, less than 7% of the fluorescence intensity was retained in tissues processed for the original uDISCO protocol, whereas for a-uDISCO, after immersion in pH-adjusted BABB-D4 (*pH* = 9.0–9.5) for 3 days, ~80% of the mean fluorescence intensity had survived.

Because the clearing protocol for hemispheres is different from the protocol for brain slices, we further assessed the fluorescence preservation of a-uDISCO in hemispheres by comparing our results with those obtained using the original uDISCO. We imaged cleared hemispheres of mouse brains with a light-sheet microscope (Supplementary Video 1). The fluorescence images were obtained immediately after clearing (i.e., 0 d) with a-uDISCO or uDISCO, and transverse projections of hemispheres and high-magnification images were obtained at the indicated brain regions as shown in Figure 3A. The results indicated that a-uDISCO fluorescence was notably brighter than that of uDISCO and allowed the visualization of finer structures, such as dendrites and axons.

Moreover, to evaluate the fluorescence changes that occurred during long-term storage, we acquired high-magnification images of cleared brains at the following time points after clearing: 0, 7, 14, 28, and 42 d. The fluorescence images of the hippocampus obtained at the four time points qualitatively showed that fluorescence was maintained for a longer time in a-uDISCO than uDISCO (Figure 3B). Furthermore, we calculated the fluorescence levels at each time point and found that the SBR was significantly higher for a-uDISCO than uDISCO during long-term storage (Figure 3C).

### a-uDISCO retains good clearing performance

As described above, a-uDISCO achieved better fluorescence preservation than did uDISCO by modifying the pH condition of the clearing agents. Whether this modification influenced clearing performance remained to be investigated.

First, we cleared whole adult mouse brains with a-uDISCO and uDISCO. We obtained bright-field images (Figure 4A) and measured the light transmittance (400 to 800 nm) of cleared whole brains (Figure 4C). The transparency of the whole brains cleared with the two methods was similar, and their transmittance spectra were very close, indicating that a-uDISCO possessed a good clearing capability similar to that of uDISCO.

We next measured changes in the sizes of whole brains after clearing. The shrinkage values along three axes, including ventral to dorsal, anterior to posterior and left to right, were calculated. We found that a-uDISCO reduced the sizes of whole brains in all three directions up to ~65% and that there was no significant difference between uDISCO and a-uDISCO (Figure 4D).

In addition, we imaged individual microglia on the dorsal surfaces of whole mouse brains both before and after clearing via a-uDISCO to evaluate the maintenance of cell morphology. Cell morphology was highly similar before and after clearing (Figure 4E). We also imaged dendritic spines on pyramidal neurons located on the surface of whole mouse brains before and after clearing via a-uDISCO to evaluate the maintenance of fine structures (Supplementary Figure 1). The morphologies of the dendritic spines were similar before and after clearing. Thus, a-uDISCO maintained fine structures well.

### a-uDISCO allows 3D visualization of whole mouse brains

**Figure 5:**
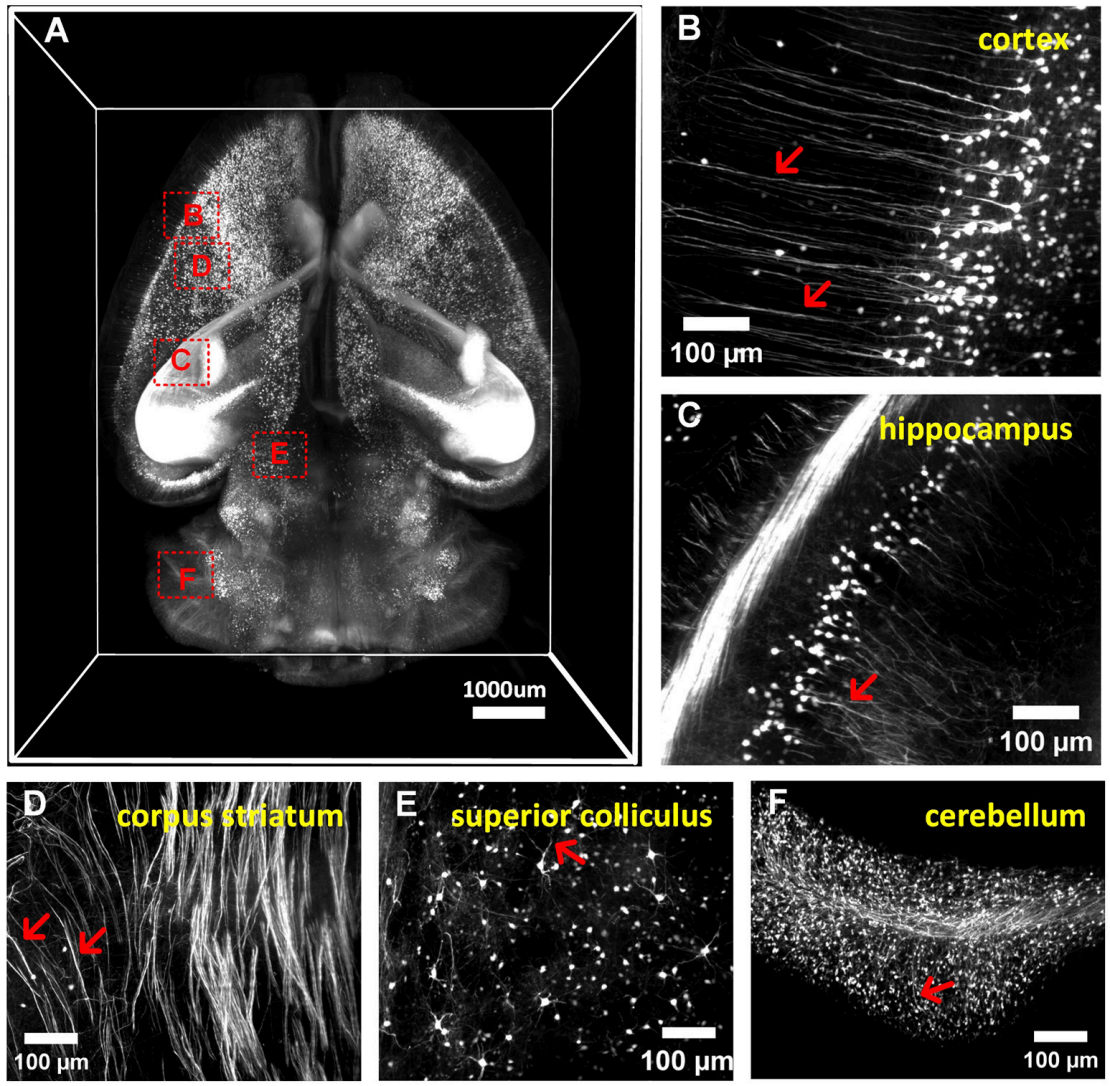
3D imaging of adult mouse brains cleared with a-uDISCO (*Thy1*-GFP-M). **(A)** 3D reconstruction of neuronal structures in the whole brain. **(B–F)** High-magnification MIP images of the boxed regions indicated in **(A)** demonstrating that neuronal structures could be visualized at a subcellular resolution in the cortex **(B)**, hippocampus **(C)**, corpus striatum **(D)**, superior colliculus **(E)**, and cerebellum **(F)**. The arrow indicates fine branches of neurons.

We conducted whole-brain (*Thy1*-GFP-M) clearing and imaging by combining a-uDISCO with a light-sheet microscope, as shown in (Figure 5A and Supplementary Video 2). We observed the details of neuronal structures in the cortex, hippocampus, corpus striatum, superior colliculus and cerebellum (Figures 5B–F). We were able to readily identify individual neurons and their extensions, including apical dendrites extending to the cerebral surface and fine axonal projections to the midbrain. a-uDISCO enables 3D imaging and the reconstruction of brain-wide neuronal structures with high resolution and could potentially be applied in connectomics studies.

## Discussion

In this study, we developed an optimized clearing protocol based on uDISCO that we have called a-uDISCO. a-uDISCO provides higher fluorescence intensity while retaining the advantages, such as high transparency and size reduction, of uDISCO. This is essential for performing whole-brain imaging by a light-sheet microscope.

Among all clearing methods, organic-solvent-based clearing methods achieve higher levels of transparency in a shorter time than other clearing methods (Richardson and Lichtman, 2015; Pan et al., 2016). As a typical organic-solvent-based clearing method, 3DISCO takes only 3 days to render a whole mouse brain transparent and can maintain fluorescent protein emission for 1–2 days (Ertürk et al., 2012). uDISCO, a subsequently developed method, preserves endogenous fluorescence for months by replacing the tetrahydrofuran with tert-butanol during the dehydration step and adding diphenyl ether in BABB clearing solution (Pan et al., 2016). Though the fluorescence preservation achieved by uDISCO is relatively higher than that of 3DISCO, it is also associated with increased background fluorescence (Lai et al., 2017). When encountering samples with low-level fluorescence protein expression, the ability of uDISCO to preserve fluorescence is not good enough. In addition, for archiving purpose and repetitive revisiting of rare samples, it also requires lower fluorescence quenching during long-term storage. Hence, achieving improved fluorescence preservation in clearing protocols was one motivation for performing this study.

Based on the pH sensitivity of GFP luminescence, we sought to determine the optimal basic environment for the reagents in uDISCO to achieve better fluorescence preservation (Xiong et al., 2014). It is worth noting that wide-field fluorescence images of uncleared 1 mm-thick brain slices looked blurrier and brighter than the cleared ones (Figure 1), purportedly because of the strong backscattering observed in thick uncleared samples. Hence, the fluorescence intensity of uncleared samples derived from wide-field images cannot be used to calculate changes in fluorescence. While the cleared samples were transparent and had similar backscattering at different time points after clearing, the fluorescence decay observed during storage was used for primary screening of pH values (Figure 1). It should also be noted that pH values in the range of 9.0–9.5 and 10.5–11.0 both achieved better fluorescence preservation. A range of 9.0–9.5 was selected in this study because it required traces of triethylamine to be added to the reagents (e.g., 60 μl in 15 ml 100% tert-butanol) while the pH values in the 10.5–11.0 range required much more (1 ml), and this might have decreased tissue transparency. A previously published protocol, FluoClearBABB, utilizes tert-butanol as the dehydration reagent and BABB as the refractive index matching reagent and also perseveres GFP fluorescence to a great extent by maintaining a basic pH throughout the procedure (Schwarz et al., 2015). However, because it does not use DCM to degrease, this method takes 10 days to clear a whole adult mouse brain, while a-uDISCO takes only 4 days.

The optical clearing methods have been widely applied not only for brain researches, but also for applications of other tissues (Kolesova et al., 2016; Stefaniuk et al., 2016; Dubey et al., 2017; Frétaud et al., 2017; Greenbaum et al., 2017; Li et al., 2017). The optimized method proposed in this paper works for both mouse brains and other tissues, such as muscle, stomach, and lung (Supplementary Figure 4). But, the transparency is poor for heme-rich organs such as heart, liver and kidney, especially for spleen. For the heme-rich tissues, the transparency can be increased by prolonging the time of PBS perfusion or washing the samples with PBS in 37°C for several hours prior to clearing process.

This is not the first attempt at optimizing clearing procedures. For example, Epp et al. (2015) and Magliaro et al. (2016) quantitatively assessed the quality of clarification over time in terms of tissue transparency and GFP protein loss using indices similar to those used in this paper. Additionally, it should be mentioned that we only investigated GFP fluorescence preservation in the optimized method. The fluorescence-preserving performance of this protocol when using other kinds of fluorescent proteins, such as RFP, YFP, and CFP, remains to be confirmed. Except for these endogenous fluorescent proteins, other exogenous fluorescent labels, such as immunofluorescent markers and nuclear dyes, which are valuable for specific labeling, also need to be investigated in future studies.

In summary, we have developed a modified protocol based on uDISCO in which the pH values of the reagents are adjusted. This optimized optical clearing method better preserved GFP fluorescence while also retaining the advantages of uDISCO, including high transparency and size reduction. When combined with a light-sheet microscope, this protocol allows the 3D visualization of neuronal networks in the whole mouse brain and provides excellent imaging quality. This method could potentially provide a better option for imaging samples with low-level fluorescent protein expression and repetitive revisiting of rare samples.

## Data availability statement

All data generated or analyzed in this study are included in the manuscript and the supplementary files.

## Author contributions

YL and TY conceived and designed the study. YL and PW prepared the samples. YL and JX performed the imaging with cameras and microscopes, processed the images, and analyzed the data. YL made the figures. YL and TY wrote the manuscript. DZ gave valuable comments and suggestions for revising the manuscript. TY and DZ provided financial support and supervised the project. All authors contributed to manuscript revisions and read and approved the submitted version.

### Conflict of interest statement

The authors declare that the research was conducted in the absence of any commercial or financial relationships that could be construed as a potential conflict of interest.

## Supplementary material

Supplementary Figure 1 The morphology of dendritic spines in a whole mouse brain before and after clearing by a-uDISCO (*Thy1*-GFP-M). Z-stack fluorescence images of dendritic spines in a whole adult *Thy1*-GFP-M mouse brain were acquired with a confocal fluorescence microscope (LSM710, Zeiss, Germany) equipped with an alphaPlan-Apochromat 63 × /1.46 oil objective (W.D. 0.1 mm). Dendritic spines located on the surface of the uncleared mouse brain were selected for imaging. To find this structure after the clearing process, we needed to map images under a low-magnification lens, similar to the process used to image the same microglia before and after clearing.

Supplementary Figure 2 Individual microglia were imaged on the surface of the mouse brain. **(A)** A whole brain was inverted on a confocal culture dish containing the clearing agents and then placed on the objective table. It should be noted that the dish had to be covered with a lid to prevent the sample from drying out. **(B)** The images obtained under low-magnification lenses (5 × objective, 10 × objective and 20 × objective) were used as maps. In addition, we adjusted the image threshold to show the blood vessels (red arrows) to identify the same brain region before and after clearing.

Supplementary Figure 3 Methods for quantification of fluorescence intensity changes. **(A)** For measuring the mean intensity of each neuron in 1 mm-thick brain slices, the freehand selection function of ImageJ software was used to draw the outline of each neuron. **(B)** The threshold function and “analyze particles” of ImageJ software were used to select the visible cell bodies with a proper size and then measure the mean intensity of each selected cell body.

Supplementary Figure 4 a-uDISCO is applicable for clearing various tissues (*Thy1*-GFP-M). **(A)** Reflective images of spinal cord before and after clearing with a-uDISCO method. **(B)** The fluorescence images of cleared spinal cord, acquired with a light-sheet microscope. **(C)** Reflective images of intact organs (muscle, intestine, stomach, lung, heart, kidney, liver, and spleen) before and after clearing with a-uDISCO method. Grid size, 1.44 mm × 1.44 mm.

Supplementary Table 1 Reagents, times and temperatures used at each experimental step.

Supplementary Table 2 The list of reagents used in this paper.

Supplementary Table 3 Troubleshooting table.

Supplementary Video 1 Comparison of fluorescence preservation of cleared hemispheres (*Thy1*-GFP-M) via uDISCO and a-uDISCO. The images were obtained by Ultramicroscope equipped with 2.0 × objective and a macro zoom body (magnification steps from 0.63 × to 6.3 ×). The video was made with Imaris software.

Supplementary Video 2 3D reconstruction of the whole brain (*Thy1*-GFP-M) cleared with a-uDISCO. The images were obtained by Ultramicroscope equipped with 2.0 × objective and a macro zoom body (magnification steps from 0.63 × to 6.3 ×). The video was made with Imaris software.
