# Supplementary figures and images for: Optimization of GFP Fluorescence Preservation by a Modified uDISCO Clearing Protocol
Source: Front Neuroanat. 2018 Aug 15;12:67. doi: 10.3389/fnana.2018.00067 (PMC6104128; doi:10.3389/fnana.2018.00067)

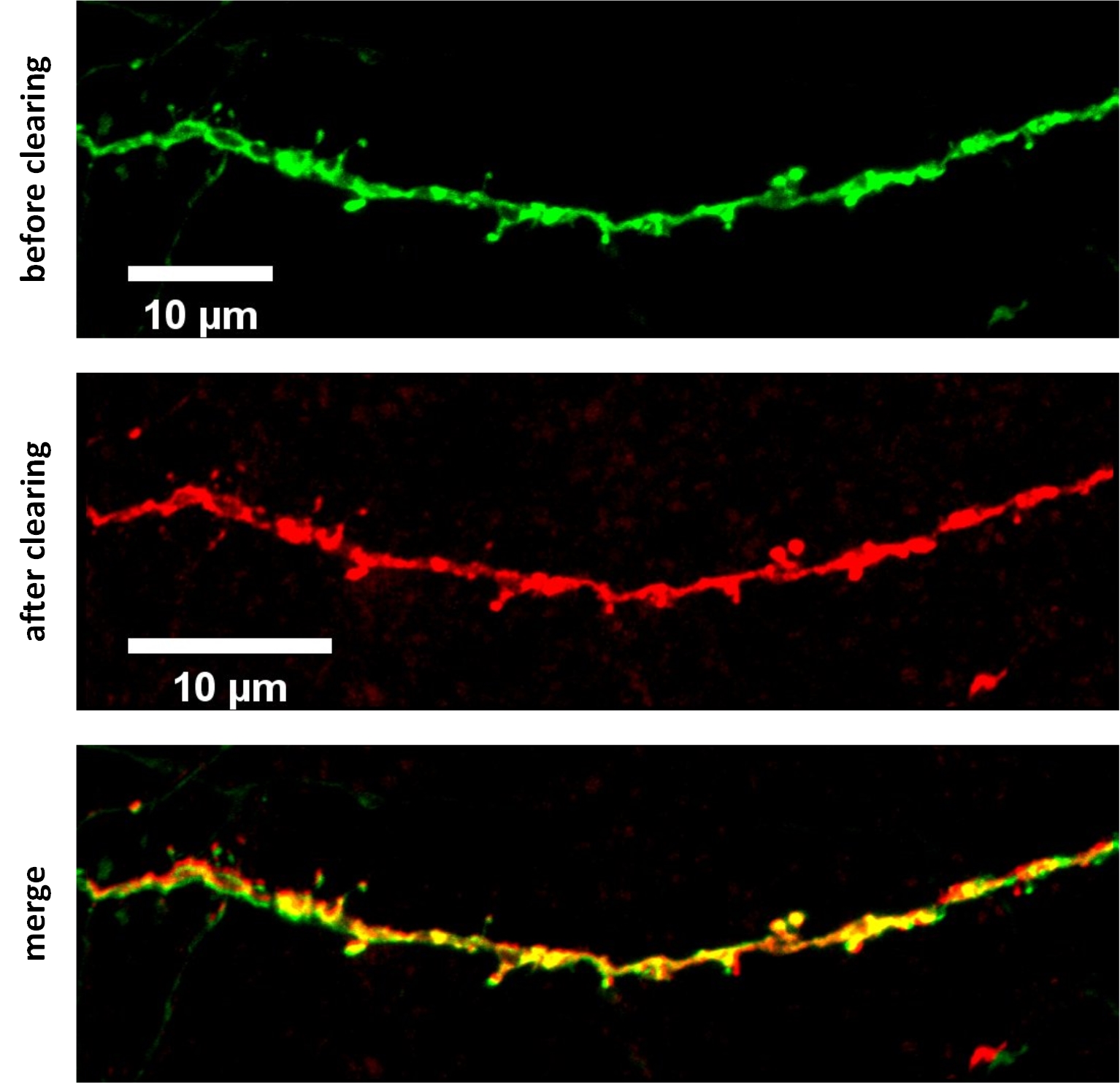

Supplement: Supplementary Figure 1 — The morphology of dendritic spines in a whole mouse brain before and after clearing by a-uDISCO (Thy1-GFP-M). Z-stack fluorescence images of dendritic spines in a whole adult Thy1-GFP-M mouse brain were acquired with a confocal fluorescence microscope (LSM710, Zeiss, Germany) equipped with an alphaPlan-Apochromat 63 × /1.46 oil objective (W.D. 0.1 mm). Dendritic spines located on the surface of the uncleared mouse brain were selected for imaging. To find this structure after the clearing process, we needed to map images under a low-magnification lens, similar to the process used to image the same microglia before and after clearing. [file Image_1.JPEG]

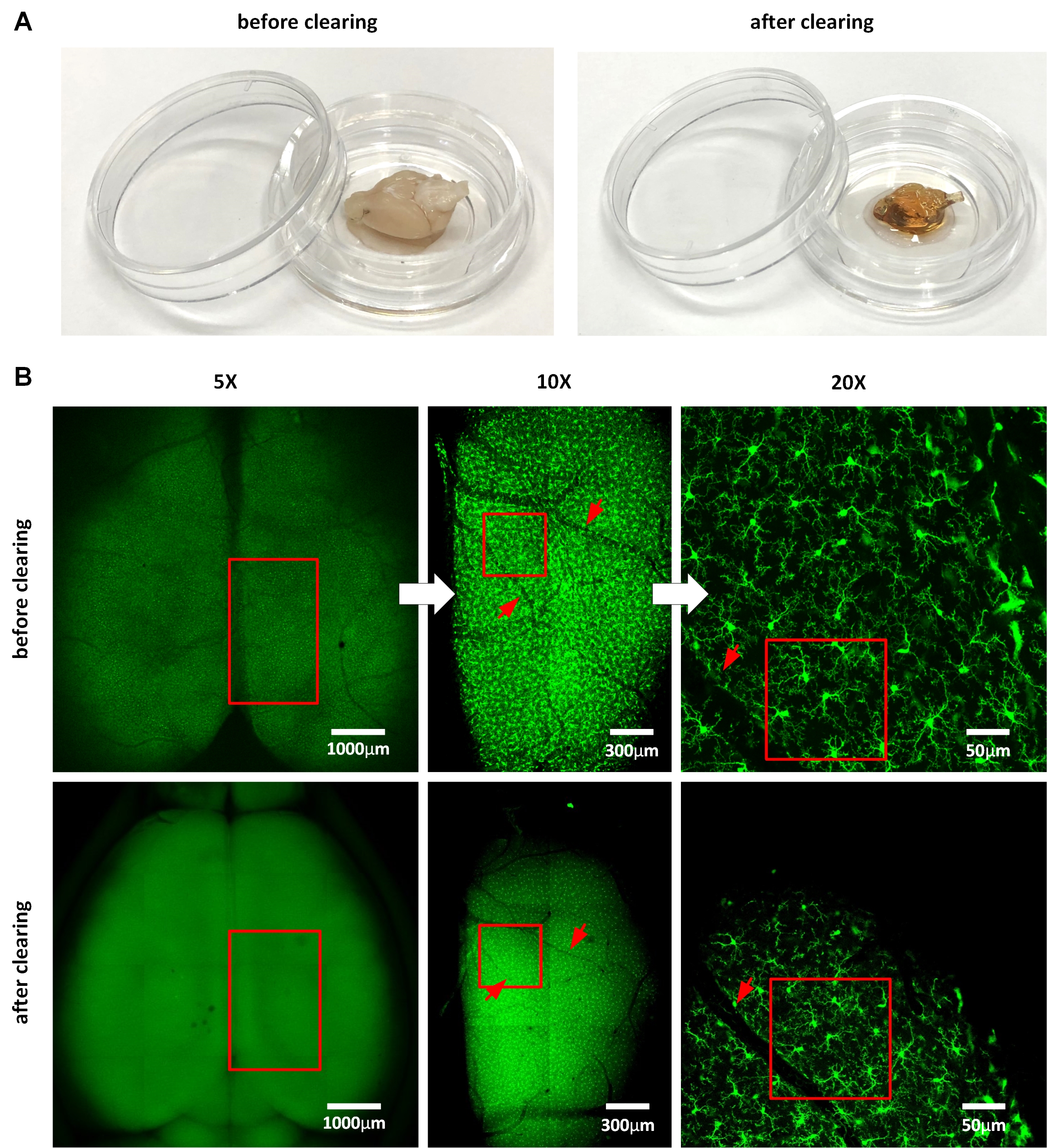

Supplement: Supplementary Figure 2 — Individual microglia were imaged on the surface of the mouse brain. (A) A whole brain was inverted on a confocal culture dish containing the clearing agents and then placed on the objective table. It should be noted that the dish had to be covered with a lid to prevent the sample from drying out. (B) The images obtained under low-magnification lenses (5 × objective, 10 × objective and 20 × objective) were used as maps. In addition, we adjusted the image threshold to show the blood vessels (red arrows) to identify the same brain region before and after clearing. [file Image_2.JPEG]

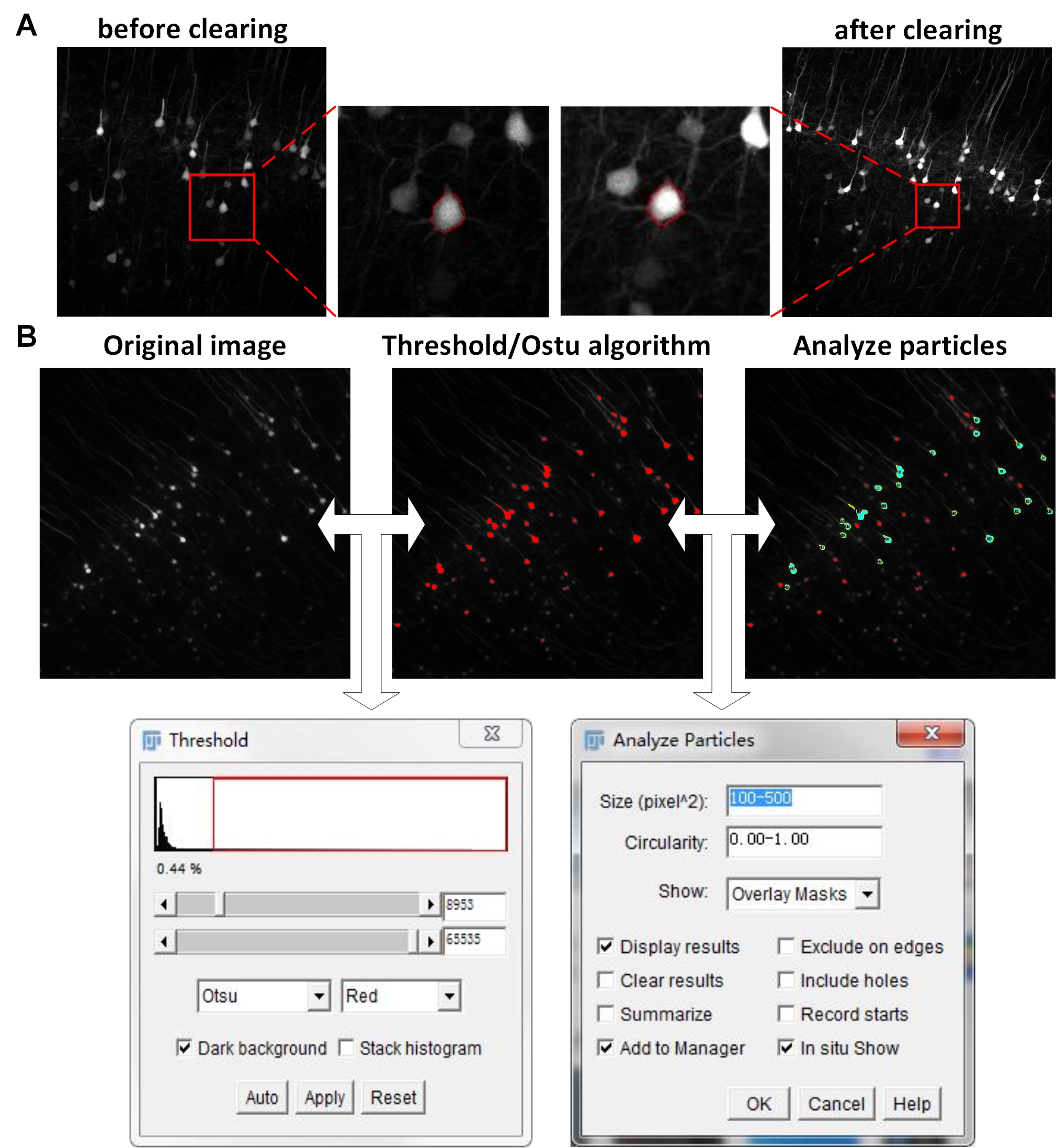

Supplement: Supplementary Figure 3 — Methods for quantification of fluorescence intensity changes. (A) For measuring the mean intensity of each neuron in 1 mm-thick brain slices, the freehand selection function of ImageJ software was used to draw the outline of each neuron. (B) The threshold function and “analyze particles” of ImageJ software were used to select the visible cell bodies with a proper size and then measure the mean intensity of each selected cell body. [file Image_3.JPEG]

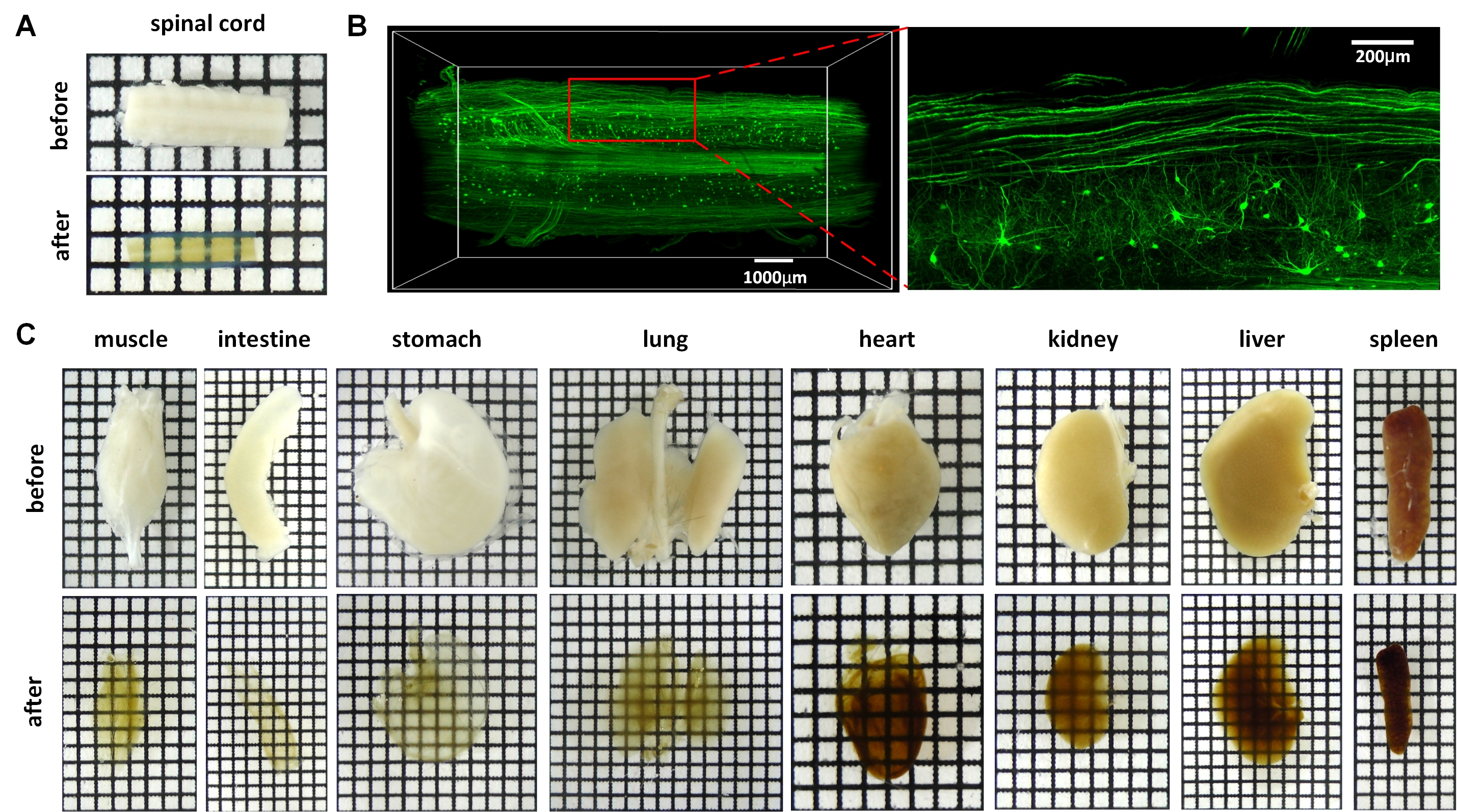

Supplement: Supplementary Figure 4 — a-uDISCO is applicable for clearing various tissues (Thy1-GFP-M). (A) Reflective images of spinal cord before and after clearing with a-uDISCO method. (B) The fluorescence images of cleared spinal cord, acquired with a light-sheet microscope. (C) Reflective images of intact organs (muscle, intestine, stomach, lung, heart, kidney, liver, and spleen) before and after clearing with a-uDISCO method. Grid size, 1.44 mm × 1.44 mm. [file Image_4.JPEG]
