# Supplementary material for: Optimization of GFP Fluorescence Preservation by a Modified uDISCO Clearing Protocol
Source: Front Neuroanat. 2018 Aug 15;12:67. doi: 10.3389/fnana.2018.00067 (PMC6104128; doi:10.3389/fnana.2018.00067)
Supplement: Supplementary Table 1 — Reagents, times and temperatures used at each experimental step. [file Table_1.docx]

Supplementary Material

Optimization of GFP fluorescence preservation by a modified uDISCO clearing protocol

Yusha Li, Jianyi Xu, Peng Wan, Tingting Yu*, Dan Zhu

* Correspondence: Tingting Yu: [yutingting@hust.edu.cn](mailto:yutingting@hust.edu.cn)

**Supplementary Table 1: Reagents, times and temperatures used at each experimental step.**

| **reagents** |  | **triethylamine added** | |  | **clearing time/temperature** | | |
| --- | --- | --- | --- | --- | --- | --- | --- |
|  |  | **uDISCO** | **a-uDISCO** |  | **brain slice** | **hemisphere** | **whole brain** |
| 30% tert-butanol |  | - | 0.5 μl/15 ml |  | 2 hrs/RT | ON/35°C | ON/35°C |
| 50% tert-butanol |  | - | 0.5 μl/15 ml |  | 2 hrs/RT | 4 hrs/35°C | 10 hrs/35°C |
| 70% tert-butanol |  | - | 0.5 μl/15 ml |  | 2 hrs/RT | 4 hrs/35°C | ON/35°C |
| 80% tert-butanol |  | - | 1 μl/15 ml |  | 2 hrs/RT | ON/35°C | 10 hrs/35°C |
| 90% tert-butanol |  | - | 3 μl/15 ml |  | 2 hrs/RT | 4 hrs/35°C | ON/35°C |
| 96% tert-butanol |  | - | 15 μl/15 ml |  | ON/RT | 4 hrs/35°C | 10 hrs/35°C |
| 100% tert-butanol |  | - | 60 μl/15 ml |  | 2 hrs/RT | ON/35°C | ON/35°C |
| DCM(+/-) |  | - | - |  | - | 45 min/RT | 60 min/RT |
| BABB-D4 |  | - | 200 μl/22.5 ml |  | 1 hr/RT | 3 hrs/RT | 5 hrs/RT |

DCM, dichloromethane; BABB-D4, benzyl alcohol: benzyl benzoate: diphenyl ether (4:8:3 in volume) + 0.4 vol% Vitamin E; RT, room temperature; -, skipped step; ON, overnight
