# Supplementary material for: Optimization of GFP Fluorescence Preservation by a Modified uDISCO Clearing Protocol
Source: Front Neuroanat. 2018 Aug 15;12:67. doi: 10.3389/fnana.2018.00067 (PMC6104128; doi:10.3389/fnana.2018.00067)
Supplement: Supplementary Table 2 — The list of reagents used in this paper. [file Table_2.docx]

Supplementary Material

Optimization of GFP fluorescence preservation by a modified uDISCO clearing protocol

Yusha Li, Jianyi Xu, Peng Wan, Tingting Yu*, Dan Zhu

*** Correspondence:** Tingting Yu: [yutingting@hust.edu.cn](mailto:yutingting@hust.edu.cn)

**Supplementary Table 2: The list of reagents used in this paper.**

| **Chemical name**  **(Cas.no.)** | **Supplier (Cat.no.)** | **Hazard statement(s)*** |
| --- | --- | --- |
| Tert-Butanol  (75-65-0) | Sigma-Aldrich (Shanghai) Trading Co. Ltd., China (360538-2L) | H225 Highly flammable liquid and vapour.  H319 Causes serious eye irritation.  H332 Harmful if inhaled.  H335 May cause respiratory irritation.  H336 May cause drowsiness or dizziness. |
| Dichloromethane  (75-09-2) | Sinopharm Chemical Reagent Co. Ltd., China (40071165) | H315 Causes skin irritation.  H319 Causes serious eye irritation.  H335 May cause respiratory irritation.  H336 May cause drowsiness or dizziness.  H351 Suspected of causing cancer.  H373 May cause damage to organs (Liver, Blood) through prolonged or repeated exposure if swallowed.  H373 May cause damage to organs (Central nervous system) through prolonged or repeated exposure if inhaled. |
| Benzyl alcohol  (100-51-6) | Sigma-Aldrich (Shanghai) Trading Co. Ltd., China (305197-1L) | H302 Harmful if swallowed.  H332 Harmful if inhaled.  H319 Causes serious eye irritation. |
| Benzyl benzoate  (120-51-4) | Sigma-Aldrich (Shanghai) Trading Co. Ltd., China (B6630-1L) | H302 Harmful if swallowed.  H411 Toxic to aquatic life with long lasting effects. |
| Diphenyl ether  (101-84-8) | Alfa Aesar (China) chemical Co. Ltd.  (A15791.30) | H319 Causes serious eye irritation.  H411 Toxic to aquatic life with long lasting effects. |
| Vitamin-E  (10191-41-0) | Alfa Aesar (China) chemical Co. Ltd.  (A17039.18) | - |
| Triethylamine  (121-44-8) | Sinopharm Chemical Reagent Co. Ltd., China (80134318) | H225 Highly flammable liquid and vapour.  H302 Harmful if swallowed.  H311 + H331 Toxic in contact with skin or if inhaled.  H314 Causes severe skin burns and eye damage.  H335 May cause respiratory irritation. |
| Phosphate buffered saline  (-) | Sigma-Aldrich (Shanghai) Trading Co. Ltd., China (P3813-10PAK) | - |
| Paraformaldehyde  (30525-89-4) | Sigma-Aldrich (Shanghai) Trading Co. Ltd., China (158127-500G) | H228 Flammable solid.  H302 + H332 Harmful if swallowed or if inhaled.  H315 Causes skin irritation.  H317 May cause an allergic skin reaction.  H318 Causes serious eye damage.  H335 May cause respiratory irritation.  H351 Suspected of causing cancer. |

*Hazard statements are referred to the Material Safety Data Sheet of Sigma-Aldrich.
