# Supplementary material for: Optimization of GFP Fluorescence Preservation by a Modified uDISCO Clearing Protocol
Source: Front Neuroanat. 2018 Aug 15;12:67. doi: 10.3389/fnana.2018.00067 (PMC6104128; doi:10.3389/fnana.2018.00067)
Supplement: Supplementary Table 3 — Troubleshooting table. [file Table_3.docx]

Supplementary Material

Optimization of GFP fluorescence preservation by a modified uDISCO clearing protocol

Yusha Li, Jianyi Xu, Peng Wan, Tingting Yu*, Dan Zhu

* Correspondence: Tingting Yu: [yutingting@hust.edu.cn](mailto:yutingting@hust.edu.cn)

**Supplementary Table 3: Troubleshooting table.**

| **Problem** | **Possible reason** | **Solution** |
| --- | --- | --- |
| The 100% tert-butanol solution is crystallized during clearing process | The room temperature is too low | Clear the samples with a 25°C water bath |
| Poor transparency of samples after clearing | Too much blood residue in the samples | Prolong the time of PBS perfusion |
| Cavity in the ventricle of a cleared brain | Bubble entered the ventricle during PBS/PFA perfusion | Check and drain the bubble in a conduit |
| Poor image quality when fluorescence imaging | Insufficient clearing | Extend the incubation time of each clearing step |
| Brain with strong spontaneous fluorescence | PFA post-fixation time is too long | Limit post-fixation time to 24 hours |
| Weak or no fluorescence signal | Negative fluorescence expression  Fluorescence quenching | Check the samples before clearing  Use freshly-prepared agents |
| Sample bleaching | Laser power is too strong | Decrease the laser power and increase the gain of the acquisition to compensate |
